# Editorial: Public-Private Partnerships as Drivers of Innovation in Healthcare

**DOI:** 10.3389/fmed.2019.00114

**Published:** 2019-05-31

**Authors:** Remco L. A. de Vrueh, Jon S. B. de Vlieger, Daan J. A. Crommelin

**Affiliations:** ^1^Lygature, Utrecht, Netherlands; ^2^Department of Pharmaceutics, Utrecht University, Utrecht, Netherlands

**Keywords:** editorial, public-private parternships, healthcare, innovation, medicine

## The format: from bilateral to multilateral

Around the turn of the century, a rather simple classification of public-private-partnerships (PPPs) in the world of medicine development sufficed. These PPPs consisted primarily of bilateral collaborations between pharmaceutical companies and academic institutes. Since then, these “simple” bilateral PPPs have been complemented by different and more diverse types of PPPs. On the one hand, PPPs emerged such as the Medicines for Malaria Venture (MMV) or the Drugs for Neglected Diseases Initiative (DNDI) with as major drivers charities, country donors, industry, and academic groups. These so-called product development partnerships (PDPs) focus on developing products for specific communicable diseases impacting health of patients in less affluent countries. On the other hand, Pharma-PPPs, such as the Innovative Medicines Initiative (IMI), emerged that focused on jointly tackling specific -precompetitive- issues in medicine development. The major players in the last category consisted of the pharmaceutical industry (large pharma), small, and medium sized enterprises (SMEs), academic institutes and–again- governmental funding programs (1, 2). Since then the background of participating stakeholders of PPPs has greatly diversified. Important new stakeholders joined the PPP consortia, including patient organizations, regulatory bodies, health technology assessment agencies, insurance companies, and IT-companies (see articles in this special issue, e.g., Aartsen et al.) All have their unique incentives to join, which makes the PPP concept more difficult to define and to evaluate in terms of its benefits. Nowadays, many PPP-flavors exist and the number and diversity continues to grow. Contributions to this special issue exemplify this current development in the PPP-world.

## Added value: in the eye of the beholder or more concrete impact measures?

**Figure 1:**
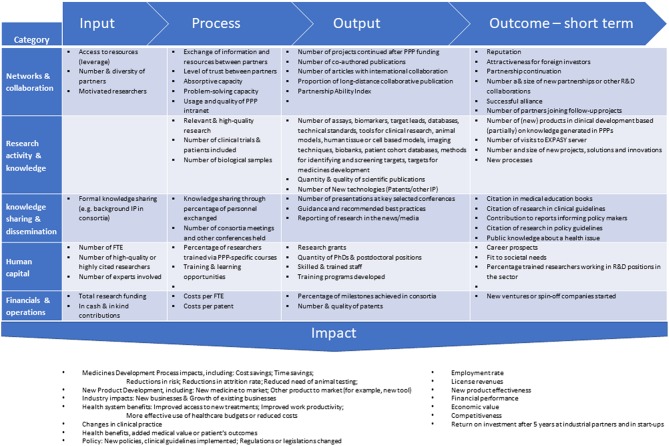
Reported performance indicators to be considered in a research PPP performance measurement system, classified into 5 categories. Figure adapted from (2).

Early on, questions were raised about the assessment of performance and success-failure of PPPs (1–3). Performance indicators to look at were identified as: the input, the process, the output, the short-term outcome, and impact. See Figure 1 for details. The basis for this methodology was already developed and tested in other fields. What makes the Pharma-PPP case so special are the long timelines–years- to measure “impact.” The classical PPP projects have a typical running time of 4–6 years. The long-term outcome-and impact e.g., in terms of concrete new medicines can only be measured many years after finishing the project and on top of that there are many “diluting” contributing factors in the post-PPP years. Moreover, simply looking at the number of medicines developed based on the activities of a PPP significantly underappreciates the additional impact from knowledge transfer, ongoing collaborations, patents, spin-off companies formed, and last but not least the educational aspect PPP initiatives offer (See Figure 1). The true impact of the first generation of PPPs now becomes visible and we can review that according to the key performance indicators set out from the start [cf. (4, 5)].

In that light, there is one question that was often raised in the early days and that can now be answered, i.e., the concern about the quality of the research output -read publications- of PPPs. Several studies made it clear (3, 4) that the impact of publications measured in terms of impact factor of scientific journals and number of citations of IMI and TI Pharma consortia was comparable–if not higher- than of articles published through “regular” academic groups efforts.

## Sustainability: to stay or to perish?

What is the chance for a consortium to survive after finishing the first funding round? Before answering this question it should be clear whether the project, topic-wise, is supposed to be continued at all? Some projects simply do not have a horizon beyond their running time. They are set up to solve a particular -often concrete- problem. However, what if a prolonged existence is foreseen? Experience teaches us that then already in an early stage the question of sustainability should be addressed. For instance, in case infrastructure has been built up, such as databanks or test facilities, further strategies to continue activities after the first funding round should be subject of discussion early on. The article by Aartsen et al. in this special issue discusses various sustainability strategies developed for IMI projects in detail and lists “lessons learned.”

## Evolution: PPP Quo Vadis?

The adoption of the “open innovation model” by the pharmaceutical industry has given the PPP concept a big push. Originally, the public partners were mainly academic and national or international public funding organizations. The large pharmaceutical industry with or without SMEs took care of the private side. Over time, the background of stakeholders in PPP consortia has diversified. Patient organizations and health insurance companies joined the consortia. Regulatory bodies such as EMA and FDA are becoming partners as well, although these institutions are very cautious to safeguard their independence from large pharma and other private stakeholders. Big IT organizations such as Google and Amazon (cloud-computing services) expanded the spectrum on the private side (Moreno et al.) as did medical device-diagnostics companies such as Siemens, Agilent, and Philips in the context of IMI. This expanding source of partners will change the character of PPP consortia. Also, the scope of activities evolved. As partners in first PPPs were jointly exploring science and collaboration in a truly pre-competitive field, a shift toward projects where partners share their strategic assets is now observed. E.g., in the IMI—European Lead Factory (see this issue: Karawajczyk et al.) industry decided to share some proprietary assets allowing competitors and public partners to boost their drug discovery programs. It demonstrates that the PPP concept has become a trusted way of working and partners now seem comfortable to evolve the model with activities closer to their core business.

These recent developments raise the question whether the original, rather narrow definitions of a PPP as mentioned at the beginning of this editorial will properly describe the PPPs in medicine development in the future. Partners outside pharma now join the game and change the dynamics and “culture.” The walls between the classical “silos” disappear rapidly.

The remaining question is then. PPP concept in the world of medicine development: Quo Vadis?

## Author Contributions

All authors listed have made a substantial, direct and intellectual contribution to the work, and approved it for publication.

### Conflict of Interest Statement

The authors declare that the research was conducted in the absence of any commercial or financial relationships that could be construed as a potential conflict of interest.
